# PLP1 Mutations in Patients with Multiple Sclerosis: Identification of a New Mutation and Potential Pathogenicity of the Mutations

**DOI:** 10.3390/jcm7100342

**Published:** 2018-10-11

**Authors:** Nancy C. Cloake, Jun Yan, Atefeh Aminian, Michael P. Pender, Judith M. Greer

**Affiliations:** 1 UQ Centre for Clinical Research, the University of Queensland, Brisbane, QLD 4029, Australia; Nancy.Cloake@qimrberghofer.edu.au (N.C.C.); j.yan@uq.edu.au (J.Y.); atefeh.aminian@yahoo.com (A.A.); 2 School of Medicine, Tehran University of Medical Sciences, Tehran 15119-43943, Iran; 3 Faculty of Medicine, the University of Queensland, Brisbane, QLD 4029, Australia; m.pender@uq.edu.au; 4 Department of Neurology, Royal Brisbane and Women’s Hospital, Brisbane, QLD 4029, Australia

**Keywords:** myelin proteolipid protein (PLP), multiple sclerosis (MS), point mutations, primary progressive MS, women, unfolded protein response, epitopes

## Abstract

*PLP1* is located on the X-chromosome and encodes myelin proteolipid protein (PLP), the most abundant protein in central nervous system myelin. Generally, point mutations in *PLP1* result in X-linked dysmyelinating disorders, such as Pelizaeus-Merzbacher disease (PMD) or spastic paraplegia type 2 (SPG2). However, several case studies have identified patients with missense point mutations in *PLP1* and clinical symptoms and signs compatible with a diagnosis of multiple sclerosis (MS). To investigate if *PLP1* mutations occur relatively frequently in MS, we sequenced the coding regions of *PLP1* in 22 female MS patients who had developed disease after the age of 40 and in 42 healthy women, and identified a missense mutation in exon 2 of *PLP1* resulting in a Leu30Val mutation in the protein in one of the MS patients. mCherry-tagged plasmids containing wild type or mutant *PLP1* sequences of PLP, including two known PMD/SPG2-related mutations as positive controls, were constructed and transfected into Cos-7 cells. In comparison with cells transfected with wild type *PLP1*, all mutations caused significant accumulation of PLP in the endoplasmic reticulum of the cells and induction of the unfolded protein response—a mechanism that leads to apoptosis of cells expressing mutant proteins. Additionally, in silico analysis of the binding of peptides containing the Leu30Val mutation to the human leukocyte antigen (HLA) molecules carried by the patient harboring this mutation suggested that the mutation could produce several novel immunogenic epitopes in this patient. These results support the idea that mutations in myelin-related genes could contribute to the development of MS in a small proportion of patients.

## 1. Introduction

Considerable debate is ongoing regarding whether multiple sclerosis (MS) is initiated in the periphery, via activation of immune cells that subsequently enter the central nervous system (CNS) and cause damage, or within the CNS via primary myelin/oligodendrocyte damage. The latter could result from mutations in molecules encoding key components of myelin or oligodendrocytes. Whereas genome-wide association studies have not suggested significant linkages to any myelin or oligodendrocyte components in MS, they have not ruled out the possibility that mutations in genes encoding these components might occur at low frequencies in MS patients. Point mutations in *PLP1*—the X chromosome gene encoding the most abundant myelin protein, myelin proteolipid protein (PLP)—occur at a low frequency in the population, and typically result in development of Pelizaeus-Merzbacher disease (PMD) or spastic paraplegia 2 (SPG2) in men, whereas female carriers are usually symptom-free [1]. However, several reports have been published of individuals with disease who meet the diagnostic criteria for MS and who carry *PLP1* point mutations [2,3]. A recent study showed that mice expressing some of these *PLP1* point mutations develop spontaneous myelin and axonal damage that is dependent on the presence of B and T lymphocytes, suggesting that *PLP1* point mutations could induce an immune-mediated disease indistinguishable from MS [4]. The above data support the idea that damage to cells within the CNS could potentially be the initiating event in some MS cases.

The exact mechanism by which these mutations could cause disease is still unknown; however, in animal models of PMD, *PLP1* mutations led to apoptosis of oligodendrocytes [5,6]. Similarly, cells transfected with *PLP1* sequences that contain known PMD mutations also undergo apoptosis due to accumulation of PLP in the endoplasmic reticulum (ER) and the induction of the unfolded protein response (UPR) [7,8,9,10]. The UPR is a cellular mechanism responsible for ensuring that incorrectly formed proteins are not expressed by cells; a clear marker of UPR activation is the translocation of the protein CCAAT/enhancer binding protein homologous protein (CHOP) from the cytoplasm of the cell to the nucleus [11,12]. In cells consistently transcribing misfolded proteins at levels difficult to overcome, the UPR initiates apoptosis in an effort to remove the defective cell [13]. It has been suggested that, at least in some MS patients, the initial pathological damage in the CNS is apoptosis of oligodendrocytes [14]. The finding of *PLP1* mutations in patients with MS-like disease therefore raises the question of whether *PLP1* mutations may induce oligodendrocyte apoptosis and be a cause of the onset of disease in some patients.

Of the two patients thus far described with *PLP1* mutations and MS, one was a 10-year-old male with relapsing-remitting MS (RR-MS) [2], and the other was a female with primary progressive MS (PP-MS) [3]. In male patients who only have one copy of the X chromosome, it is likely that any signs of disease caused by *PLP1* mutations would occur at a young age. In women, X chromosomes containing mutations are usually inactivated, but the inactivated X chromosome can change (referred to as skewing), particularly as women age [15,16]. Therefore, it is more likely that *PLP1* mutations could be a potential cause of MS symptoms in female patients who have skewed X inactivation, or who develop disease at a later age. This is of potential interest particularly for female patients with PP-MS, who typically develop disease at a later age than women who develop RR-MS [17].

In the current study, we sequenced the protein-coding exons of *PLP1* in 22 female PP-MS patients who developed MS after the age of 40. One patient was found to carry a novel *PLP1* mutation. We then assessed in vitro evidence of cell damage (altered trafficking of PLP, and activation of CHOP and the UPR) in cells transfected with plasmids containing the mutant *PLP1* sequences occurring in the patient we identified and in the two other reported MS-related *PLP1* mutation patients, in order to determine whether the mutations found in the patients were potentially pathogenic.

## 2. Experimental Methods

### 2.1. Patients and Controls

Blood samples (5 mL) were collected from 42 healthy women and from 22 female patients with PP-MS after informed consent was obtained. Demographic details of patients and controls are shown in Table 1. This study was approved by the Human Ethics Committees of the Royal Brisbane and Women’s Hospital (HREC/11/QRBW/28) and the University of Queensland, Australia (20110000711).

### 2.2. DNA Extraction and PLP1 Sequencing

DNA was extracted using a NucleoSpin^®^ Blood XL DNA extraction kit (Macherey-Nagel, Düren, Germany), according to the manufacturer’s protocol. Primers (Life Technologies, Mulgrave Australia) to amplify exons 2 to 7 of human *PLP1* were designed from the human *PLP1* mRNA sequence using the online tool National Center for Biotechnology Information (NCBI) Primer-Blast (http://www.ncbi.nlm.nih.gov/tools/primer-blast). DNA was sequenced at the Australian Genome Research Facility (AGRF), the University of Queensland, Brisbane, Australia. Sequences were analysed using Sequence Scanner v1.0 software (Life Technologies, Carlsbad, CA, USA).

### 2.3. PLP1-Containing Vectors

Plasmids containing wild type (wt) or mutant *PLP1* sequences encoding the 7 exons of PLP were constructed by the Protein Expression Facility (University of Queensland, Brisbane, Australia). Gene inserts were first cloned into the pOPIN-(n)-mCherry vector, in order to couple the inserts with the mCherry tag gene sequence to allow subsequent identification of transfected cells. Finally, the gene inserts were inserted into the pcDNA™ 3.3-TOPO TA (Life Technologies, Carlsbad, CA, USA) vector. DNA vector constructs were amplified and plasmid DNA was extracted using a MaxiPrep Plasmid DNA extraction kit (Qiagen, Hilden, Germany), as per the manufacturer’s instructions. To confirm that the sequences of the gene inserts were correct, the plasmid DNA preparations were sequenced at the Australian Genome Research Facility Ltd. (Brisbane, Australia).

### 2.4. Transfection of Cos-7 Cells

Transfection via electroporation was performed on dissociated Cos-7 cells using the Nucleofection System (Lonza, Basel, Switzerland) together with the Amaxa II transfection device (Lonza, Basel, Switzerland), following the manufacturer’s recommended protocol. The transfected cells were plated in 16-well chamber slides (Thermo Fisher, Melbourne, Australia) at a concentration of 10^4^ cells/well. Each slide included non-transfected cells and pulse-only cells as controls.

### 2.5. Immunocytochemical Analysis of Transfected Cells

Cells were fixed in 4% paraformaldehyde (Merck, Darmstadt, Germany) for 15 min at room temperature, and permeabilized for 15 min with 0.05% saponin (Sigma, St Louis, MO, USA) and 0.5% bovine serum albumin (BSA; Sigma, St Louis, MO, USA) in dH_2_O (saponin-BSA). Primary antibody (rabbit anti-PLP antibody; 1:2000; Thermo Fisher, Melbourne, Australia) or mouse anti-CHOP antibody (1:200; Abcam, Cambridge, UK) in saponin-BSA was then added for 2 h at room temperature. After 3 washes, cells were labelled with the appropriate fluorescein isothiocyanate (FITC)-conjugated secondary antibody (donkey anti-rabbit immunoglobulin (Ig), 1:300, (Santa Cruz Biotechnology, Dallas, TX, USA) or goat anti-mouse Ig (1:500; Abcam, Cambridge, UK)) in saponin-BSA solution for 2 h at room temperature in the dark. After 3 washes, the cells were then labelled with 4′,6-diamidino-2-phenylindole (DAPI) (Sigma; 1:20,000 dilution in 1.5M NaCl) for 15 min at room temperature in the dark, washed 3 times in phosphate-buffered saline (PBS), and coverslips were mounted.

To assess protein trafficking out of the ER, cells were stained for 30 min at room temperature with ER-ID Green Assay Kit (Enzo Life Sciences, Farmingdale, NY, USA) per the manufacturer’s recommended protocol. Using this protocol, the ER fluoresced green. 

Cells were photographed using an Eclipse 50i fluorescent microscope (Nikon Instruments, Tokyo, Japan) with an Olympus DP70 camera (Tokyo, Japan). Photomicrographs of multiple fields were taken using blue (to detect nuclei), red (to detect mCherry), and green (to detect FITC) filters. The photos were merged using Photoshop 7.0 (Adobe Systems, San Jose, CA, USA). Images were analyzed using Image J software.

### 2.6. Statistical Analyses

Two-way analysis of variance (ANOVA) with Dunnett’s multiple comparison test was used to compare transfection efficiency, percentages of cells trafficking PLP out of the ER, and intensity of CHOP expression.

### 2.7. HLA Binding Predictions

The human leukocyte antigen (HLA) class I and class II binding predictions were produced on November 14, 2016 using the Immune Epitope Database (IEDB—www.iedb.org) analysis resource tools [19,20,21].

## 3. Results

### 3.1. Sequencing of *PLP1* in Human Subjects

**Figure 1:**
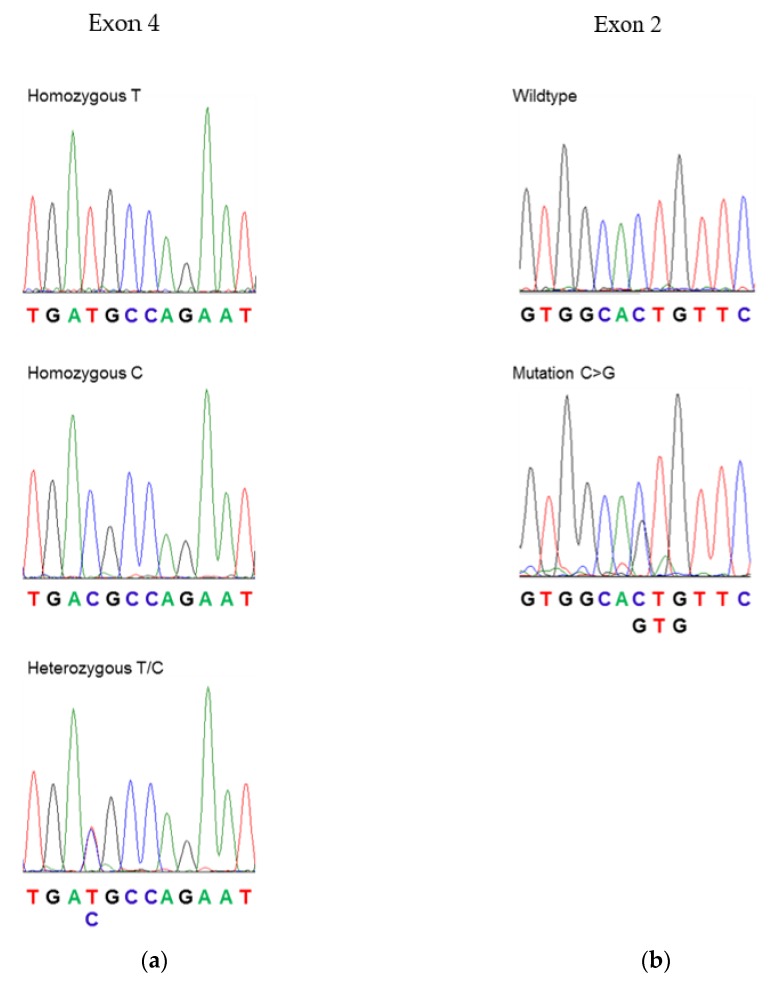
Polymorphisms/mutations in proteolipid protein (PLP): (**a**) Exon 4 polymorphism: the three panels on the left show the DNA sequences of exon 4 of individuals who are homozygous at T (top), homozygous at C (center), or heterozygous at this position. (**b**) Exon 2 mutation: the top panel shows the wildtype sequence seen in a healthy woman, and the bottom panel shows a C > G mutation found in a female patient with primary progressive multiple sclerosis (PP-MS). This mutation results in a coding change from CTG (leucine) to GTG (valine).

There is little information available regarding mutations or polymorphisms in *PLP1* in healthy individuals, aside from relatives of patients with PMD. Therefore, exons 2 to 7 of *PLP1* were sequenced in genomic DNA from a group of 42 healthy women and 22 women with PP-MS. Exon 1 was not sequenced, as it only encodes a single amino acid (methionine), which is not incorporated into the mature protein. The different genotypes of the single nucleotide polymorphism GAT > GAC (encoding the silent mutation D202D), which were previously reported in *PLP1*, occurred at approximately the same frequency in healthy control and MS patients (Figure 1a and Table 2). No other mutations were observed in the healthy control group. However, one PP-MS patient had a mutation in exon 2 (CTG > GTG), which would result in a coding change from leucine to valine at amino acid 30 (L30V) (Figure 1b). This patient presented with a history of gradually increasing right lower limb weakness for 10 years and a burning sensation in the left lower limb for several years. Neurological examination revealed bilateral lower limb weakness, pathologically brisk right knee jerk, bilateral extensor plantar responses, and decreased pain and temperature sensation in the left T7 to S5 dermatomes. Magnetic resonance imaging of the brain and cervical spine demonstrated multiple deep cerebral white matter lesions, as well as lesions in the medulla and upper cervical spinal cord. She had oligoclonal immunoglobulin G (IgG) bands restricted to the cerebrospinal fluid and met the 2010 Revised McDonald Criteria for a diagnosis of MS [22]. She had one daughter; however, analysis of the daughter’s DNA showed that she did not carry the exon 2 mutation.

### 3.2. Effects of Point Mutations in *PLP1* on PLP Expression in Cos-7 Cells

**Figure 2:**
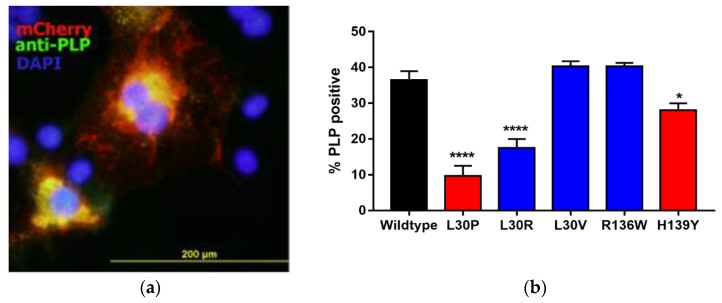
Expression of PLP in Cos-7 cells. (**a**) Anti-PLP antibody co-localizes with expression of mCherry in transfected Cos-7 cells after 24 h in culture. (**b**) Percentages of cells transfected with the different *PLP1* constructs that expressed PLP after 48 h in culture. Bars show mean ± SE of four experiments. The black bar shows cells transfected with the wildtype construct, the red bars are those transfected with constructs based on known Pelizaeus-Merzbacher disease/spastic paraplegia type 2 (PMD/SPG2) mutations, and the blue bars are cells transfected with constructs containing the MS-related mutations. * *p* < 0.05, **** *p* < 0.0001.

We used a Cos-7 cell transfection/expression system to assess the effects of *PLP1* mutations on overall cell health and trafficking of PLP through the cells, as previously described [23]. Constructs were prepared that carried *PLP1*, one of the five mutations listed in Table 3, or an empty vector, coupled to a mCherry tag to allow identification of transfected cells. Cells were transfected into Cos-7 cells. Initial studies were completed to show that mCherry expression co-localised with anti-PLP antibody staining (Figure 2a). Thereafter, the number of cells that were mCherry positive, compared to the number of nuclei present, was used to assess the number of transfected cells 48 h after transfection (Figure 2b). Approximately 40% of cells transfected with the tagged-wtPLP vector expressed PLP by 48 h after transfection (Figure 2b). In comparison, there were significantly fewer PLP+ cells in the cultures transfected with the PMD/SPG2-related *PLP1* mutant sequences, L30P, or H139Y. Of the three MS-associated mutations tested, only the L30R mutation caused a significant reduction in the percentage of PLP+ cells. This variation in expression was not due to effects on gene transcription, as analysis of mRNA from these cells using quantitative real-time polymerase chain reaction (qRT-PCR) showed no significant differences in any of the cells expressing mutant *PLP1* compared to the wild type (data not shown).

### 3.3. Effects of Point Mutations in *PLP1* on the Trafficking Pattern of PLP in Cos-7 Cells

**Figure 3:**
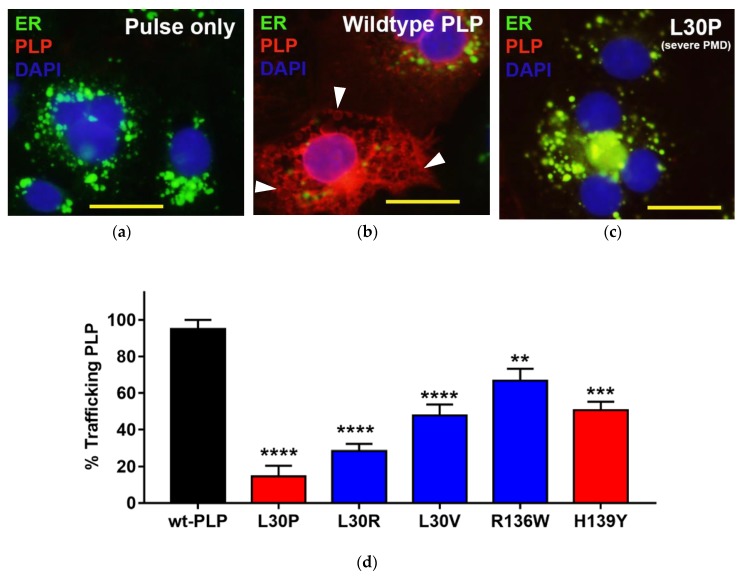
Mutations at L30 cause PLP to be retained within the endoplasmic reticulum (ER). (**a**) Cells transfected with empty vector show labelling with the ER-ID stain (green), but no labelling for PLP, as expected. (**b**) After 24 h of culture of cells transfected with the wild-type (wt)-PLP vector, mCherry-tagged PLP (red) trafficked out of the ER. (**c**) In contrast, almost all PLP was retained within the ER (indicated by co-localisation of PLP and ER labelling) in cells transfected with the L30P mutant. Blue = 4′,6-diamidino-2-phenylindole (DAPI), red = mCherry-tagged PLP, and green = ER-ID stain. (Size bar = 50 µm). (**d**) Summary of the percentage of cells expressing the different *PLP1* mutations that can traffic PLP out of the ER. The bars show mean ± SE of at least 20 cells analyzed for each group, and are color-coded as in Figure 2b. ** *p* < 0.01, *** *p* < 0.001, **** *p* < 0.0001.

Cos-7 cells transfected with *PLP1* sequences containing PMD-associated point mutations were previously reported to accumulate PLP in the endoplasmic reticulum (ER) and it does not traffic out to the cell membrane [7]. Cos-7 cells transfected with mCherry-tagged wt- or mutant-*PLP1* were therefore investigated for the distribution of PLP within the cells by co-labelling cells with ER-ID to show the ER (Figure 3a). At 24 h post-transfection, almost all cells transfected with wt-*PLP1* expressed mCherry throughout the cells (Figure 3b,d). Some of the staining outside of the ER had a vesicular appearance (arrowheads in Figure 3b), consistent with previous reports [8]. The percentage of PLP-expressing cells in which PLP had moved out from the ER and/or showed vesicular staining was also determined in cells transfected with the various *PLP1* mutants. Compared to wt-*PLP1* transfectants, the amount of PLP that moved out of the ER in cells transfected with all of the mutant *PLP1* sequences significantly decreased, although the transfectants having the greatest effect on transport out of the ER were those with mutations at L30, particularly L30P (Figure 3c,d).

### 3.4. Investigation of the Induction of the Unfolded Protein Response in *PLP1*-Transfected Cos-7 Cells

The accumulation of mutant PLP in the ER of an oligodendrocyte leads to the activation of the UPR, a natural feedback system that is in place to help cells clear incorrectly synthesised proteins. Upon UPR activation, CHOP expression is upregulated and ER-localised CHOP translocates to the nucleus to activate genes involved in UPR regulation [26,27,28]. We therefore investigated CHOP expression as a measure of induction of the UPR by the *PLP1* mutants.

**Figure 4:**
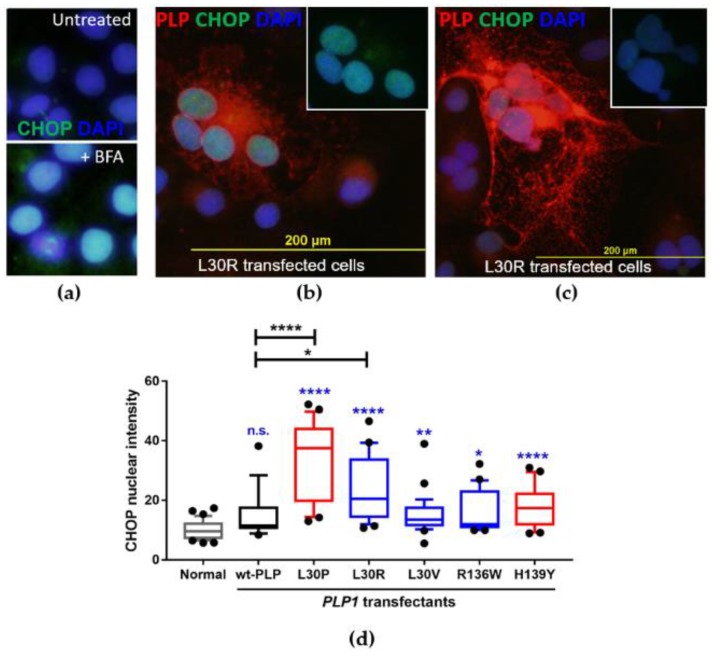
Induction of the unfolded protein response (UPR), as indicated by increased CCAAT/enhancer binding protein homologous protein (CHOP) expression in the nuclei following Brefeldin A (BFA) treatment. (**a**) CHOP staining in untreated (no BFA) cells and Cos-7 cells treated with BFA for 6 h. Nuclei are stained blue with DAPI. In the untreated cells, the level of BFA staining was very low. In the treated cells, the overall level of CHOP expression (green) was higher, and there was a concentration of CHOP in the nuclei. (**b**) Examples of CHOP accumulation in L30R transfected cells in which PLP was been retained in the ER (inset shows just CHOP and DAPI overlay). (**c**) CHOP did not accumulate in L30R transfected cells in which PLP was able to move out into the cell periphery. (**d**) Summary of the CHOP nuclear intensity in transfected Cos-7 cells 24 h after transfection. Bars show the interquartile range and whiskers the 10th–90th percentile of CHOP staining in the nuclei of at least 20 cells per transfection. Asterisks/text in blue above each bar show *p*-value compared to normal Cos-7 cells. n.s.: not significant, * *p* < 0.05, ** *p* < 0.01, and **** *p* < 0.0001.

As a positive control for the study of CHOP expression and activation of the UPR in our Cos-7 system, cells were incubated for 6 h with 1:1000 Brefeldin A (BFA, eBiosciences, San Diego, CA, USA), as prolonged exposure of cells to BFA was previously shown to activate the UPR [28]. All cells upregulated CHOP in the nuclei following exposure to BFA (Figure 4a). Cos-7 cells transfected with the *PLP1* mutants and cultured for 24 h were also assessed for upregulation of nuclear CHOP. Compared with normal untransfected Cos-7 cells, there was no significant increase in CHOP intensity for pulsed (no DNA control; data not shown) or wild-type PLP transfected cells. As anticipated from our trafficking pattern study, L30P- and L30R-transfected cells showed the most significant increase in CHOP expression in the nucleus compared to both normal cells (*p* < 0.0001) and in cells transfected with wt-*PLP1* (Figure 4). In these cells, the intensity of nuclear CHOP expression was clearly linked to the ability of the cell to traffic PLP out of the ER. Cells that were unable to traffic PLP out of the ER (Figure 4b) had a much higher intensity of nuclear CHOP staining than did cells expressing PLP throughout the whole cell (Figure 4c), indicating that protein accumulation and activation of the UPR is associated with a failure of cells to traffic PLP. CHOP nuclear expression in L30V-, R136W-, and H139Y-transfected cells also significantly increased compared with normal cells, but L30V-, R136W-, and H139Y-transfected cells were not significantly different compared with cells transfected with wt-*PLP1* (Figure 4d).

### 3.5. L30V Mutation Could Potentially Change Immune Responsiveness to PLP

The above results suggest that the L30V mutation identified in the patient in our study had a relatively mild effect on PLP expression and trafficking. Even if *PLP1* mutations were not sufficiently severe to cause widespread apoptosis of oligodendrocytes in patients, another way in which they could potentially be related to the development of MS would be to change the ability of the peptide to bind to the HLA molecules carried by the patient, thereby changing a non-immunogenic PLP peptide to an immunogenic and potentially pathogenic epitope. The HLA type of this patient was HLA-A2, 29; B44; DRB1*07:01, 13:02; DQA1*01:02, 02:01; DQB1*03:03, 06:04. For each of the HLA-A, HLA-B, and HLA-DRB1 alleles, in silico analysis was used to investigate the potential binding of PLP peptides containing residue 30 as either a leucine or valine, using the IEDB analysis resource (www.iedb.org) [19,20,21]. The 50% concentrations of peptides with either L or V at residue 30 that were predicted to inhibit binding of a control peptide to the HLA allele of interest (the half maximal inhibitory concentration (IC_50_) values) are shown in Table 4. The lower the number in the table, the stronger the binding, with peptides with an IC_50_ less than one predicted to be very high binders. Those with an IC_50_ between 1 and 10 being moderate binders, and those between 10 and 50 being relatively weak binders. In most cases, the predicted binding of a peptide with either L30 or V30 was similar for any specific HLA allele, which is not surprising as L→V is a relatively conservative substitution. However, for some peptides, a change from L30 to V30 would significantly increase the binding of the peptide to a HLA molecule. For example, peptide FFGVALFCG was predicted to be an extremely weak binder to DRB1*07:01 (IC_50_ > 50 µM), whereas FFGVAVFCG was predicted to bind with extremely high affinity to that allele (IC_50_ = 0.9 µM), and would therefore have a much higher potential to generate an immune response in the patient.

## 4. Discussion

**Figure 5:**
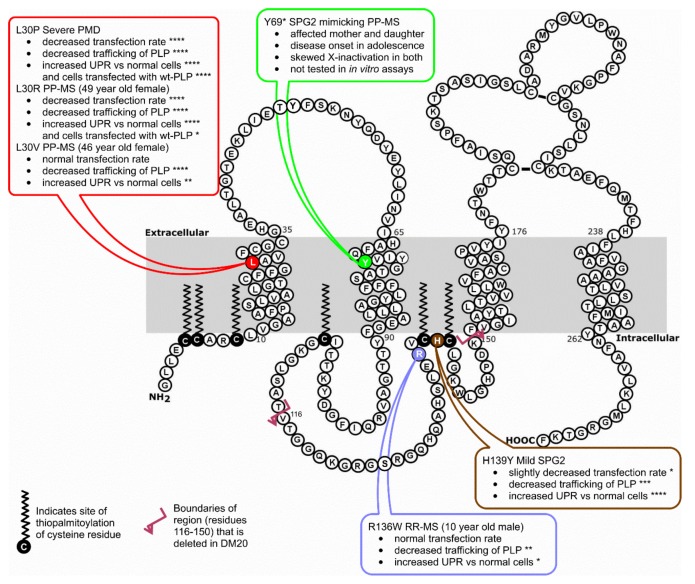
Diagram showing the orientation of PLP in the myelin membrane, the location of the mutations mentioned in this study, and, for those mutations tested in this study, a summary of their effects on Cos-7 cells, with the asterisks indicating the significance of the effects (* *p* < 0.05, ** *p* < 0.01, *** *p* < 0.001, **** *p* < 0.0001).

The initiating events that lead to MS are still unknown, and there has been considerable debate on the relative merits of “outside-in” (i.e., autoimmune) vs. “inside-out” (i.e., primary CNS damage) models for development of MS [29]. Multiple genes, mostly of low penetrance, have been linked to susceptibility to the development of MS, and the nature of those genes strongly suggests that autoimmunity drives the development of disease in the majority of patients [30,31]. However, not all patients follow the same pathway to disease development. In the current study, we identified a female patient with disease meeting the diagnostic criteria for PP-MS who carried a point mutation in *PLP1* that resulted in an amino acid substitution at residue 30 of PLP (L30V). We subsequently showed that this and another two *PLP1* mutations that were described in people who meet the criteria for MS [2,3] were all able (to varying degrees) to affect trafficking of PLP out of the ER and induce the UPR (Figure 5). These findings suggest that these *PLP1* mutations would all have the potential to cause damage to oligodendrocytes, and that they could therefore represent an alternative pathway for MS development. This conclusion is supported by the recent finding that mice carrying the L30R and R136W mutations show neuroinflammation and clinically relevant axonal degeneration, neuronal loss, and brain atrophy by one year of age [4]. In those mice, inactivation of the recombination activating gene 1, which is necessary for maturation of B and T lymphocytes, resulted in a less severe clinical phenotype, suggesting that the adaptive immune response plays an important role in the disease development, similar to what is observed in MS.

The region of the *PLP1* gene encoding residue 30 appears to be a hotspot for mutation, as not only did it feature in the new patient identified in our study, but also in one of the other previously described MS patients (L30R mutation) [3]. The L30P mutation has also been reported in cases of severe PMD [24]. The concept of mutation hotspots in *PLP1* has been previously described in PMD patients, and there are several amino acids at which multiple mutations have been reported [1,32]. The L30V mutation identified in our study (which, of the three mutations at L30, showed the fewest effects on PLP expression or trafficking or on induction of the UPR) is a conservative mutation that would not be expected to have dramatic effects on the hydrophobicity of the first transmembrane domain in which it resides, as both L and V are hydrophobic amino acids with similar structures and neutral side chains. In contrast, an L30R mutation in the first transmembrane domain of PLP would be expected to change the hydrophobicity, overall charge, and/or secondary structure of the transmembrane helix, and could have a deleterious effect on the membrane-spanning potential of the helix and greatly disrupt the PLP structure. The L30P substitution would result in a rigid bend being imposed on the polypeptide and would be expected to affect the secondary structure of the transmembrane helix.

The patients carrying the L30R and L30V mutations were both women and both developed clinical signs and symptoms consistent with PP-MS at an older age. The late onset of clinical symptoms and the progressive nature of their disease could potentially be explained by the dynamics of X-inactivation in women. Female carriers of transmembrane and extracellular *PLP1* PMD mutations, which tend to cause the most severe cases of PMD in men, have been shown to be less likely to show any clinical manifestations of disease than women carrying mutations in the intracellular domains of PLP [1,3]. This is likely due to skewed X inactivation of the mutant X chromosome, as it has previously been reported that expression of X-linked genes in women can favor the gene on one X chromosome over another in order to avoid producing mutated proteins [15]. However, there is also evidence that suggests that a woman’s X-inactivation pattern can change over time, even in the presence of mutant genes [33]. One study reported that women with PP-MS exhibit a greater degree of X-inactivation skewing than healthy women and those with other MS subtypes [34]. These findings, taken together, could be one explanation for the late onset of neurological symptoms seen in the patients with L30R and L30V mutations. We predict that if *PLP1* mutations are a potential initiating factor in MS, then female patients who develop disease at an older age would be the most likely female patients in whom such mutations would be found. However, there is another recent report of two women (mother and daughter) who both carried a novel nonsense *PLP1* mutation at codon 210 T > G [35] (Figure 5). Although disease in these patients mimicked PP-MS, clinical signs of disease were present from adolescence in both. Of interest, there was evidence of skewing of X inactivation in both patients, supporting the idea that heterozygous *PLP1* mutations in women can lead to disease if skewed X inactivation occurs, regardless of whether this occurs due to aging or to some other process.

Since *PLP1* is located on the X chromosome, it is likely that any men with *PLP1* mutations would develop disease at a very early age. The nature of the mutation would determine the clinical phenotype of the disease (i.e., PMD, SPG2, or MS-like). The R136W mutation [2] occurs in exon 3B, which is deleted in the alternatively-spliced isoform of PLP known as DM20. Thus the 10-year-old boy who carried this mutation would be able to produce normal DM20. DM20 is expressed prior to PLP during ontogenesis and may have a role in the development of new oligodendrocytes [36,37]; however, in post-natal CNS, the ratio changes and PLP becomes more abundant than DM20 (PLP:DM20 ratio 3:1 in the CNS) [38], with the presence of both isoforms critical for successful myelination in adolescence [39]. In in vitro studies, the presence of the correct DM20 sequence was shown to help mutant PLP traffic out of the ER [40].

In the 24 or 48 h in vitro assays in this paper, the L30V and R136W variants showed fewer detrimental effects on the transfected Cos-7 cells than the L30R mutation. However, both the L30R and R136W mutations, when expressed as transgenes in mice, resulted in significant damage within the CNS. Thus, over time, even mild mutations such as R136W can cause sufficient oligodendrocyte damage so that disease ensues. Since the severity of the in vitro effects seen in cells expressing the L30V mutation lay between that seen in cells expressing the L30R and the R136W mutations, the L30V mutation identified in the current study could likely result in damage within the CNS.

Direct oligodendrocyte damage might not be the only way in which *PLP1* mutations could induce MS. As shown in Table 4, several peptides containing the L30V mutation were predicted to bind with higher affinity to some of the HLA molecules carried by the patient than the native peptide, thus creating de novo epitopes and potentially inducing/activating a new set of autoreactive T cells in this patient. The generation of such responses would be dependent on the presence of specific proteases in the patient that would allow processing of the peptides shown in Table 3, and on the presence of T cells in the patient’s T cell repertoire that could recognize the new epitopes.

The results of this study support the idea that mutations in myelin-related genes could contribute to the development of MS in a small proportion of patients. Furthermore, they suggest two possible mechanisms by which *PLP1* mutations could cause MS: *PLP1* mutations could drive an inside-out disease process by directly damaging oligodendrocytes; however, they could initiate an outside-in process, causing the expression of neoantigens that could be targeted by the immune system. Both processes could occur simultaneously. Genes on the X chromosome have been somewhat ignored in genetic studies that have been undertaken in MS, but *PLP1* should possibly be considered for further investigation, particularly when MS develops in juvenile men or in older women.

## Figures and Tables

**Table 1:** Demographics of female primary progressive multiple sclerosis (PP-MS) patients and controls.

Group	Number	Mean Age (Years ± SE)	Mean MS Duration (Years ± SE)	EDSS ^1^
Healthy controls	42	37.8 ± 1.8	Not applicable	Not applicable
PP-MS	22	53.0 ± 2.7	7.8 ± 1.5	6.3 ± 0.3

^1^ EDSS: Expanded Disability Status Scale, with scores ranging from 0 (no neurological impairment) to 10 (death from MS) [18]; SE: standard error.

**Table 2:** Frequency of *PLP1* polymorphisms in female patients with primary progressive multiple sclerosis (PP-MS) and in healthy female controls.

Group	Polymorphism			
	Exon 4–D202D			Other Exons
	T/T	C/T	C/C	
PP-MS (*n* = 22)	12 (54.5%)	7 (31.8%)	3 (13.6%)	1 (L30V) het ^1^
Healthy (*n* = 42)	24 (57.0%)	16 (38.1%)	2 (4.8%)	None

^1^ het = heterozygous.

**Table 3:** *PLP1* constructs used in the study.

Designation	Mutation		Description
	Codon	Amino Acid Change	
Wildtype	None	None	Wildtype PLP sequence
L30P	89T > C	Leucine to proline at position 30	Severe PMD mutation [24]
L30R	89T > G	Leucine to arginine at position 30	MS-like disease mutation [3]
L30V	88C > G	Leucine to valine at position 30	MS-like disease (this study)
R136W	406C > T	Arginine to tryptophan at position 136	MS-like disease mutation [2]
H139Y	415C > T	Histidine to tyrosine at position 139	Mild SPG2 mutation [25] ^1^

MS: multiple sclerosis; PLP: myelin proteolipid protein; PMD: Pelizaeus-Merzbacher disease; SPG2: spastic paraplegia type 2; ^1^ This mutation was chosen because it is the closest mutation to residue 136 known for *PLP1*.

**Table 4:** Predicted half maximal inhibitory concentration (IC_50_) (µM) of potential peptides from the region surrounding residue 30 of PLP for binding to the HLA-DR, HLA-A, and HLA-B alleles carried by the patient when residue 30 is an L or a V.

Starting Amino Acid	Peptide	DRB1*07:01		DRB1*13:02		A*02		A*29		B*44	
		L	V	L	V	L	V	L	V	L	V
22	GLCFFGVA(L/V)	**0.6**	– ^1^	8.7	5.8	0.4	0.2	9.2	8.5	–	–
23	LCFFGVA(L/V)F	0.5	0.5	15.7	14.4	33.2	9.2	1.4	6.3	12.4	18.4
24	CFFGVA(L/V)FC	–	–	12.9	11.8	17.6	7.2	0.5	1.8	15.7	24.7
25	FFGVA(L/V)FCG	–	**0.9**	–	–	14.7	14.3	6.8	7.6	–	–
26	FGVA(L/V)FCGC	–	–	–	–	21.7	10.2	–	–	–	–
27	GVA(L/V)FCGCG	–	–	–	–	–	**24.7**	14.5	15.4	–	–
28	VA(L/V)FCGCGH	1.8	3.7	–	–	–	**29.5**	11.1	14.9	–	–
29	A(L/V)FCGCGHE	–	–	–	–	16.7	26.8	15.2	11.2	–	–
30	(L/V)FCGCGHEA	–	–	10.5	7.8	7.7	19.9	3.2	3.4	–	–

^1^ indicates a very weak binding affinity of >50 µM.
